# Parental Education and Unmet Therapeutic Needs Among School-Aged Children With Developmental Delays: A Pooled Cross-Sectional Survey in Aachen, Germany

**DOI:** 10.3389/ijph.2025.1608050

**Published:** 2025-02-20

**Authors:** Timo-Kolja Pförtner, Sabine Deisz, Simone Köster, Monika Gube

**Affiliations:** ^1^ Research Methods Division, Faculty of Human Sciences, University of Cologne, Cologne, Germany; ^2^ Health Authority of the City and Area of Aachen, Aachen, Germany

**Keywords:** parental education, preschool children, unmet therapy need, developmental delay, school entry

## Abstract

**Objectives:**

Early intervention in the context of developmental delays is crucial for mitigating the adverse effects of developmental delays. The purpose of this study was to determine inequalities in the unmet therapy needs of school-aged children with developmental delays by parental education.

**Methods:**

Data from the 2015–2019 school-entry survey of Aachen, Germany, were used (N = 7,211). We assessed unmet therapy needs by parental education for global developmental delays and for delays in physical coordination, selective attention, visual-motor skills, visual perception and reasoning, knowledge of numbers and quantities, and speech and language.

**Results:**

Inequalities in unmet therapy needs were identified across all domains to the disadvantage of children with low parental education. Significant disparities in unmet therapy needs were found for global developmental delay and for delays in physical coordination, selective attention, visual‒motor skills, and speech and language.

**Conclusion:**

Unmet therapy needs affect children with lower parental education more frequently across all areas of developmental delay, highlighting the need for further studies and interventions to explain and reduce disparities in the unmet therapy needs of children with developmental delays.

## Introduction

Childhood serves as a formative period during which fundamental skills and capacities are acquired. It encompasses a multidimensional process of physical (e.g., motor skills, sensory perception), cognitive (e.g., problem-solving, language acquisition), emotional (e.g., regulation of emotions, interpersonal relationships), and social development (e.g., interactions with others, empathy) [1]. Optimal development in early childhood is associated with improved physical health, cognitive abilities, emotional functioning, and social competencies [2]. Individuals who experience successful childhood development are more likely to achieve academic success, build positive social relationships, and cope with life challenges with resilience [3]. In contrast, developmental delays can hinder these processes, affecting key areas, such as motor skills, language, cognition, and behavior, leading to poorer academic careers, less meaningful relationships, increased mental health problems, and a diminished quality of life [4]. The repercussions of developmental delays often extend beyond childhood, negatively influencing long-term outcomes in education, employment, and health over the life course [5]. These long-term effects highlight the public health significance of developmental delays.

The reversibility of developmental delays largely depends on early identification and timely intervention. Early developmental screenings, coupled with targeted therapies and support systems, are crucial for improving outcomes [6].

However, meeting professional treatment often depends on a myriad of factors, including parental socioeconomic status (SES). Parental SES refers to the social and economic factors that determine a family’s position within society, typically measured through a combination of income, education, and occupation. Parental SES is strongly associated with health outcomes and developmental delays, as evidenced by a social gradient, where individuals from lower socioeconomic backgrounds consistently experience poorer health and developmental outcomes. Initial research indicates that higher parental SES is a facilitator and that lower parental SES is a barrier to services access of pre-school children [7–9]. For example, McMannus et al. [10], using retrospective data from the USA on children under 35 months with diagnosed conditions or developmental delays, reported that low-income children were 13.6% less likely to receive physical therapy and 10.4% less likely to receive occupational therapy than higher-income children were.

Explanations for these socioeconomic disparities in unmet therapy needs for developmental delays often focus on factors influencing access to healthcare services. According to the conceptual framework of Levesque et al., access to care is a multidimensional concept associated with both user characteristics (ability to perceive needs, ability to seek, reach, and pay care, and ability to engage) and features of the healthcare system (approachability, acceptability, availability and accommodation, affordability, and appropriateness) [11]. Each factor on both sides may contribute to socioeconomic inequalities in unmet therapy needs for children with developmental delays. On the demand side, research indicates disparities in the ability to perceive developmental delays, seek care, or reach care, often disadvantaging children from lower SES families [7, 12–14]. (On the supply side, studies have shown that families with lower SES face a lack of services, less effective outreach, and costs for services, all of which may contribute to inequalities in unmet therapy needs for children with developmental delays [14, 15].

Given the initial evidence of socioeconomic disparities in access to therapeutic services for children with developmental delays, this study aims to identify whether and to what extent inequalities exist in unmet therapy needs among school-aged children through parental education. Previous research has focused predominantly on very young children or included broad age ranges, leaving the specific needs of school-aged children insufficiently addressed. By concentrating on this age group, the present study contributes to the growing body of evidence on existing inequalities in care and expands this understanding by examining the need for professional treatment across different domains of developmental delay. Using data from the school-entry examination (SEE) in Aachen, Germany, from 2015–2019, the analyses explored unmet therapy needs in various developmental delay areas by parental education, including physical coordination, selective attention, visual‒motor skills, visual perception, knowledge of numbers and quantities, and speech and language.

## Methods

### Setting and Population

The analyses are based on the mandatory SEE conducted between 2015 and 2019 in the Aachen region of Germany. The Aachen region is located in the federal state of North Rhine-Westphalia and is considered one of the poorest regions in Germany [16]. The region does not show disproportionately more diagnoses of developmental delays than the average at the state level. This is also evident for unmet therapy needs in various developmental delay domains, as a comparison of official statistics from the state of North Rhine-Westphalia with school entry examinations in the Aachen region suggests. In Germany, the healthcare system ensures universal coverage through statutory health insurance, which covers approximately 90% of the population and provides comprehensive care funded through income-based contributions, and private health insurance, which offers faster access and flexibility for high earners and the self-employed. Child care is equally structured, with government-subsidized options, such as Kindertagesstätte (Kita) for children under 6, including kindergartens for ages 3–6, and childminders (Tagespflege), for more flexible care. Every child over 1 year old has a legal right to child care, ensuring affordability and availability for families. In addition to a range of early prevention services in Germany, such as prenatal classes, parent-child groups, services for family centers, home visiting programs or early help for special needs, the Aachen region is engaged in family and child interventions in the local setting, such as in the “Family-Friendly Community” program, the program “Children in Focus” or the “Aachen Family Alliance.” All these services are freely available and aimed directly and indirectly at promoting children’s development.

SEE is an obligatory assessment in Germany. In most of the federal states, including North Rhine-Westphalia, SEE takes place in the year before school starts at the age 5–6 years [17]. School entry examinations aim 1) to examine the child’s physical, mental, and health development for the demands of everyday school life; 2) to identify disabilities, developmental delays, or special needs; 3) to offer guidance for diagnostic and therapeutic interventions, along with recommendations for organizing everyday school life; and 4) to provide advice on selecting the appropriate type of school.

The Aachen public health department administers school entry examinations in Aachen, Germany, approximately twelve to two months before school starts. Each annual school enrollment cohort in Aachen consists of approximately 4,800 children, predominantly aged 5–6 years. The school entry survey was utilized in this study because it provides a full population-based sample, thereby minimizing bias from selective participation commonly encountered in voluntary surveys. This comprehensive dataset ensures more representative and reliable insights into the developmental status and therapeutic needs of children at school entry.

### Dependent Variable

Unmet therapy needs for developmental delays were assessed on the basis of child development evaluations of physical coordination, selective attention, visual‒motor skills, visual perception, knowledge of numbers and quantities, and speech and language skills. Assessments of these domains included the results of standardized diagnostic screenings as well as qualitative assessments conducted by a school physician according to the social-pediatric screening (SOPESS) [18]. The data recorded whether children with identified developmental delays were currently receiving treatment, but they did not include an evaluation of whether therapeutic intervention was deemed necessary. On the basis of this information, the following analyses distinguish between children with met therapy needs (value 0) and those with unmet therapy needs (value 1). In addition, global developmental delay, in which children show delays in at least two domains of development, was also considered [19]. A distinction was made between children with met therapy needs in fewer than two domains of developmental delay (value 0) and those with unmet therapy needs in two or more domains of delay (value 1).

### Independent Variable

The measurement of parental education relies on the voluntary information provided by legal guardians regarding their schooling and vocational training. This information was transformed into an index ranging from 1 to 8 [20]. Using the index values, guardians were categorized into those with low educational status (values of 1–3), intermediate educational status (values of 4–6), and high educational status (values of 7–8). In the case of single-parent households, the educational status of the available parent or guardian was used. When data from both parents were available, the highest educational level was considered to reflect the household’s overall socioeconomic status.

### Covariates

The control variables included in the analysis were selected to address potential confounders related to educational inequalities in unmet therapeutic needs among children with developmental delays. These variables were as follows: child’s age, gender, and primary language spoken at home during the first four years of life (German or other), which may influence access to and utilization of health services; family structure (two birth parents, single birth parent, birth parent with partner, orphanage care, or other arrangements), reflecting socioeconomic and caregiving contexts that can affect developmental support; paediatric child development examinations (categorized as all examinations completed, not all completed, or screening booklet missing), which indicate access to preventive health services; and kindergarten attendance (yes or no), representing early childhood educational participation, a key factor in developmental support and intervention access.

### Analytical Sample

The analytical sample comprises all children with a delay in child development and for whom complete information on the indicators considered in this study was available during the observation period (n = 7,211) (Table 1). Approximately 8% (n = 622) of the children with a diagnosed developmental delay had to be excluded from the analyses because of missing data (ranging from 7% to 9%).

**TABLE 1:** Characteristics of preschool children in the sample from the school entry surveys (School-entry survey, Aachen region, Germany 2015–2019).

	Overall	Parental education		
		Low	Intermediate	High
Overall n (%)	7,211 (100.0)	2,566 (35.6)	2,841 (39.4)	1,804 (25.0)
Gender n (%)				
Male	4,485 (62.2)	1,500 (58.5)	1,815 (63.9)	1,170 (64.9)
Female	2,726 (37.8)	1,066 (41.5)	1,026 (36.1)	634 (35.1)
Age n (%)				
5 years	4,675 (64.8)	1,724 (67.2)	1,831 (64.4)	1,120 (62.1)
6 years	2,536 (35.2)	842 (32.8)	1,010 (35.6)	684 (37.9)
Family structure n (%)				
Natural parents	5,344 (74.1)	1,626 (63.4)	2,159 (76.0)	1,559 (86.4)
Single parent	1,236 (17.1)	630 (24.5)	446 (15.7)	160 (8.9)
Natural parent with partner	381 (5.3)	189 (7.4)	161 (5.7)	31 (1.7)
Children’s home/Caregivers	122 (1.7)	35 (1.4)	50 (1.8)	37 (2.1)
Others	128 (1.8)	86 (3.3)	25 (0.9)	17 (0.9)
Pediatric child development examinations n (%)				
All realized	5,011 (69.5)	1,525 (59.4)	2,190 (77.1)	1,296 (71.8)
Not all realized	1,590 (22.0)	733 (28.6)	481 (16.9)	376 (20.8)
Missing screening booklet	610 (8.5)	308 (12.0)	170 (6.0)	132 (7.3)
Kindergarten ever attended n (%)				
Yes	6,618 (91.8)	2,271 (88.5)	2,656 (93.5)	1,691 (93.7)
No	593 (8.2)	295 (11.5)	185 (6.5)	113 (6.3)
Language in the first 4 years of life n (%)				
German	4,113 (57.0)	1,211 (47.2)	1,801 (63.4)	1,101 (61.0)
Other	3,098 (43.0)	1,355 (52.8)	1,040 (36.6)	703 (39.0)
Domain of developmental delay n (%)				
Physical coordination	1,161 (16.1)	425 (16.6)	459 (16.2)	277 (15.4)
Selective attention	1,051 (14.6)	476 (18.7)	386 (13.6)	186 (10.3)
Visual-motor skills	2,692 (37.3)	1,149 (44.8)	1,041 (36.6)	502 (27.3)
Visual perception and reasoning	876 (12.1)	475 (18.5)	283 (10.0)	118 (6.5)
Knowledge of numbers and quantities	679 (9.4)	392 (15.3)	206 (7.3)	86 (4.8)
Speech and language	5,746 (79.7)	2,084 (81.2)	2,245 (79.0)	1,417 (78.5)
Global developmental delay^a^ n (%)	2,698 (37.4)	1,234 (48.1)	1,001 (35.2)	463 (25.7)
Number of unmet therapy needs M (SD)	0.73 (1.03)	1.03 (1.23)	0.62 (0.90)	0.47 (0.76)
Survey year n (%)				
2015	1,283 (17.8)	444 (17.3)	498 (17.5)	341 (18.9)
2016	1,328 (18.4)	502 (19.6)	505 (17.8)	321 (17.8)
2017	1,443 (20.0)	512 (20.0)	583 (20.5)	348 (19.3)
2018	1,555 (21.6)	548 (21.4)	656 (23.1)	351 (19.5)
2019	1,602 (22.2)	560 (21.8)	599 (21.1)	443 (24.6)

Note: n, number of observations. M, Mean. SD, Standard deviation.

^a^ Global developmental delay = delay in two or more domains of development.

### Statistical Analyses

In the first step, absolute and relative frequencies for all categorical variables were presented for the entire sample and stratified by parental education. In the second step, unmet therapy needs across various domains of developmental delays, including global developmental delays, were illustrated for the total sample and by parental education. Additionally, we performed bivariate logistic regression modelling to detect significant differences in unmet therapy needs based on parental education levels. In the third step, stepwise logistic regression modelling was conducted to examine the robustness of the association between unmet therapy needs and parental education. Model 1 included adjustments for parental education and the year of the school entry examination to account for the primary independent variable of interest and temporal variations that could affect therapeutic needs. Model 2 expanded upon Model 1 by introducing individual-level variables (age and sex), which are directly associated with developmental milestones, alongside family-level variables (family structure) to capture caregiving dynamics that may shape access to health and social resources. Model 3 was built on Model 2 by adding primary language spoken at home in the first four years of life, reflecting the potential impact of the language environment on developmental outcomes and service access. Finally, Model 4 incorporated preventive child development check-ups and kindergarten attendance, key factors related to healthcare utilization and early educational support, to fully adjust for influences on unmet therapeutic needs linked to healthcare engagement and early intervention opportunities.

The degree of model fit was assessed with McFadden’s pseudo R^2^ for the logistic regression models, which ranged from 0 to 1, with higher values indicating a better model fit. Analyses were performed with Stata SE 18.0 (StataCorp, Texas, United States).

## Results

Table 1 presents the composition of the analytical sample of 7,211 children with developmental delays. Among them, 35.6% had low parental education, 39.4% had medium parental education, and 25.0% had high parental education. The proportion of boys with developmental delays was consistently greater across all educational groups, with the disparity becoming more pronounced at higher parental education levels. Most children were under six years old and lived with both biological parents, although children from less-educated households were more likely to live in single-parent families. While most children completed all recommended pediatric child development examinations, 28.6% with low parental education missed at least one, whereas 16.9% and 20.8% with medium and high education, respectively, did. Over 90% attended kindergarten, but nonattendance was greater among children from less-educated families. More than half spoke German during their first four years, although a non-German home language was more common in families with lower education. Delays in visual‒motor skills and speech and language were most common. Developmental delays and the number of unmet therapy needs were more prevalent as parental education decreased, with diagnosed delays increasing over time.

**FIGURE 1:**
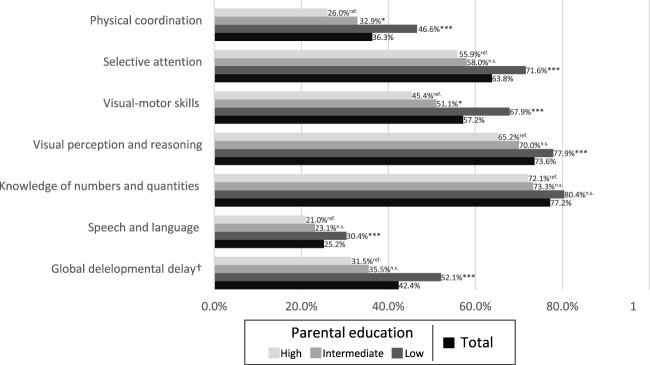
Unmet therapy needs of children with developmental delay in total and by parental education. Bivariate logistic regression modeling was performed with high parental education as reference group (School-entry survey, Aachen region, Germany 2015–2019). Notes: ***p < .001, **p < .01, *p < .05, n.s. = not significant.

Figure 1 shows the percentage of children with unmet therapy needs for developmental delays overall and by parental education. Children with delays in visual perception and reasoning, knowledge of numbers and quantities, and selective attention were most often untreated. In contrast, only 25.2% of the children with speech and language delays did not receive treatment. Additionally, 42.4% of the children with global developmental delays were untreated for at least two delays.

A clear social gradient is evident, with rates of unmet therapy needs increasing as parental education decreases (see Figure 1). For example, 26.0% of children with high parental education had unmet therapy needs for physical coordination delays, compared with 32.9% with intermediate education and 46.6% with low parental education. Significant ( p <0.05) differences in unmet therapy needs exist, particularly between children with high and low parental education levels across all domains of child development, except for delays in knowledge of numbers and quantities. The strongest inequality in unmet therapy needs emerged for delays in visual‒motor skills, physical coordination, selective attention, and global developmental delay.

Table 2 displays the stepwise logistic regression results for the associations between parental education and unmet therapy needs for single developmental delays and global developmental delays. The multivariate regressions largely confirm the descriptive findings: children with lower parental education levels have the highest odds of unmet therapy needs. This association is particularly strong for delays in physical coordination, visual‒motor skills, and global developmental delays. In contrast, unmet therapy needs did not significantly differ between children with intermediate and high parental education levels in all domains of child development. Moreover, no significant differences in unmet therapy needs by parental education were found for delays in visual perception and reasoning or knowledge of numbers and quantities. Overall, the results did not substantially change when individual-level and family-level variables in Model 2, primary language spoken at home in the first four years of life in Model 3, and care-relevant variables in Model 4 were additionally considered in the models.

**TABLE 2:** Logistic regression of unmet therapy needs in various domains of developmental delays by parental education (School-entry survey, Aachen region, Germany 2015–2019).

	Unmet therapy needs in:	Physical coordination	Selective attention	Visual-motor skills	Visual perception and reasoning	Knowledge of numbers and quantities	Speech and language	Global developmental delay
		OR (CI 95%)	OR (CI 95%)	OR (CI 95%)	OR (CI 95%)	OR (CI 95%)	OR (CI 95%)	OR (CI 95%)
M1	Parental education (ref.: high)							
	Intermediate	1.44* (1.03; 2.02)	1.09 (0.76; 1.55)	1.27* (1.02; 1.57)	1.21 (0.76; 1.94)	1.11 (0.62; 2.02)	1.13 (0.96; 1.33)	1.19 (0.94; 1.50)
	Low	2.63*** (1.88; 3.68)	1.92*** (1.38; 2.79)	2.54*** (2.05; 3.15)	1.80*** (1.15; 2.81)	1.75* (1.00; 3.08)	1.64*** (1.40; 1.93)	2.33*** (1.86; 2.92)
	R^2^	0.051	0.020	0.033	0.045	0.077	0.011	0.032
M2	Parental education (ref.: high)							
	Intermediate	1.37 (0.97; 1.93)	1.06 (0.74; 1.52)	1.215 (0.98; 1.51)	1.14 (0.70; 1.84)	1.12 (0.61; 2.06)	1.13 (0.96; 1.33)	1.17 (0.92; 1.48)
	Low	2.30*** (1.62; 3.26)	1.91*** (1.33; 2.74)	2.28*** (1.82; 2.85)	1.58 (0.99; 2.53)	1.67 (0.93; 3.00)	1.59*** (1.35; 1.87)	2.20*** (1.74; 2.78)
	R^2^	0.069	0.023	0.044	0.061	0.090	0.022	0.039
M3	Parental education (ref.: high)							
	Intermediate	1.38 (0.98; 1.94)	1.08 (0.75; 1.55)	1.21 (0.97; 1.51)	1.19 (0.73; 1.94)	1.16 (0.63; 2.14)	1.13 (0.96; 1.33)	1.20 (0.94; 1.52)
	Low	2.25*** (1.58; 3.20)	1.82*** (1.26; 2.63)	2.16*** (1.72; 2.71)	1.51 (0.94; 2.44)	1.70 (0.95; 3.08)	1.55*** (1.31; 1.83)	2.15*** (1.70; 2.72)
	R^2^	0.070	0.031	0.049	0.079	0.095	0.024	0.044
M4	Parental education (ref.: high)							
	Intermediate	1.39 (0.98; 1.97)	1.08 (0.75; 1.56)	1.25* (1.00; 1.56)	1.26 (0.77; 2.07)	1.11 (0.60; 2.06)	1.17 (0.99; 1.38)	1.25 (0.98; 1.60)
	Low	2.14*** (1.50; 3.05)	1.66*** (1.14; 2.40)	2.06*** (1.64; 2.58)	1.46 (0.90; 2.37)	1.50 (0.8; 2.74)	1.49*** (1.26; 1.76)	2.04*** (1.61; 2.59)
	R^2^	0.084	0.048	0.063	0.093	0.108	0.036	0.063
N		1,161	1,051	2,692	876	679	5,746	2,698

Note: OR, Odds Ratio; CI, Confidence Interval; M1, parental education + survey wave; M2, M1 + age, gender, and family structure; M3 = M2 + primary language spoken at home in the first four years of life; M4, M3 + pediatric child development examinations and kindergarten attendance. ***p < .001, **p < .01, *p < .05.

## Discussion

This study investigated the association between parental education and the unmet therapy needs of preschool children with developmental delays via anonymized data from SEE in the region of Aachen, Germany. First, the results revealed high rates of unmet therapy needs in children, with variation across different domains of child development. Second, this study identified inequalities in the unmet therapy needs of children with developmental delays caused by parental education to the disadvantage of children with low parental education. These inequalities were observed across all domains, with a significant inequality in the unmet therapy need for global developmental delay, as well as for delays in physical coordination, selective attention, visual‒motor skills, and speech and language.

The results revealed that a significant number of developmental delays were not treated by the time of the school entry examination. The proportion of unmet therapy needs varied by type of developmental delay, ranging from 25.5% for speech and language delays to more than 70.0% for delays in selective attention, visual perception and reasoning, and knowledge of numbers and quantities. It is important to note that the data from school entry medical examinations do not indicate whether a developmental delay needs treatment. Some untreated developmental delays may therefore not require any treatment. However, earlier studies also pointed to unmet therapy needs for children with developmental delays and attributed this to various factors [21]. There may have been a period during which the developmental delay was undetected and untreated, with the condition being diagnosed only at the time of the school-entry examination. For example, research indicates that while autism spectrum disorder can be diagnosed as early as 24 months, a significant number of children do not receive their diagnosis until they reach school age [22]. A lack of diagnostic procedures or a lack of attention by physicians combined with parents’ limited knowledge of developmental milestones can contribute to developmental delays remaining undetected and untreated for a certain time [23]. Even after a diagnosis has been made, some parents may refuse treatment because either they do not accept the diagnosis or they are skeptical about the effectiveness of the treatment [24, 25]. Moreover, the use of services for the treatment of developmental delays by parents can be perceived as stigmatizing [26, 27]. In addition to individual factors, structural barriers within the healthcare system—such as a lack of available providers, long waiting lists, or transportation problems—can also account for the observed results [28].

The results also revealed structural deprivation in the unmet therapy needs of children with low parental education. Children with low parental education consistently lacked treatment across all areas of developmental delay examined in this study. Significant differences in unmet therapy needs were observed for global developmental delays, as well as for delays in physical coordination, selective attention, visual‒motor skills, and language and speech. The disparities in unmet therapy needs identified in this study align with findings from previous research [7–9]. For example, it is well established that higher parental education is correlated with a greater likelihood of attending pediatric child development examinations and early interventions [29].

To address this prevention dilemma, as described by Ulrich et al. [29], addressing barriers to access to care associated with both user characteristics and features of the healthcare system is crucial. At the level of user characteristics, low parental education is linked to limited financial resources, which in turn is associated with lower utilization of healthcare services [30]. Additionally, lower parental education often correlates with reduced health literacy, limited knowledge about child development and care, and lower levels of parental engagement in their child’s care, which may contribute to the observed disparities in unmet therapy needs [31–33]. Psychosocial factors, such as lower levels of social support and higher rates of mental health issues among parents with lower education levels, may further explain the increased levels of unmet therapy needed for developmental delays in children with low parental education [34, 35].

The features of the healthcare system may further explain the disparities in unmet therapy needs associated with parental education. For example, Greiner et al. reported that walking distance to primary care in Germany is greater for individuals living in deprived areas [31]. Schillen et al. [36] reported significantly lower coverage of primary care and pediatric services in socially disadvantaged areas of a German city. As parents with lower educational levels are more likely to live in disadvantaged areas, these geographic barriers to access may contribute to disparities in unmet therapy needs. Finally, studies from Germany have also shown longer waiting times for general practitioners and specialists among lower-income groups [37]. Longer waiting times may also affect parents with lower educational levels, contributing to the unmet therapeutic needs of children with developmental delays. Inequalities in the delivery of care can further contribute to disparities in unmet therapy needs, especially when referrals to specialists are not made or when recommended treatments are declined owing to a lack of trust. For example, weak family‒professional partnerships contribute to the association between low parental education and poor ratings of child- and family-focused services of children with autism [38]. However, further studies are needed to explore the association between parental education and the unmet therapy needs of children with developmental delays, considering both the user characteristics and features of the healthcare system.

### Policy and Practice

The findings of this study have several important practical implications for policymakers, healthcare providers, and educators aiming to reduce disparities in unmet therapy needs among children with developmental delays. First, the results highlight the necessity of (re-)designing targeted interventions specifically aimed at families with low parental education. This might also imply a perspective change from “hardly reaching” families towards thinking about what makes the service that is offered hard to accept for a particular family [39]. Families need to understand the role of services and what they can provide. Therefore, dissonance in communication, such as cultural, language and literacy barriers or difficulties in accessing information about existing services or in asking for help, needs to be addressed and adapted. Research shows that parents are more likely to use services when they are designed to meet their needs, address their concerns, and fit their everyday lives [40].

Second, to address structural barriers to service access, expanding pediatric services in underserved areas, reducing waiting times for appointments, and improving transportation options for families with limited access to services are essential. This may also include an improvement of a more welcoming and less intimidating atmosphere to reduce perceptions of stigmatization and anxiety for families [39]. Anticipating and addressing potential perceptions of stigmatization by services can play a crucial role in effectively engaging less educated families in utilizing services [41]. Addressing additional potential structural barriers, such as high staff turnover, a lack of effort, inconsistency, a perceived lack of resources or poor quality of service, might further improve access to and use of services by low-educated families [39]. The study also emphasized that many developmental delays are not diagnosed until the school entry examination, suggesting that early screening programs could have a significant impact. Integrating developmental screening into routine pediatric visits, particularly in socioeconomically disadvantaged communities, could enable earlier diagnosis and intervention.

### Future Research

The findings of this study suggest several avenues for future research. Further investigations are needed to explore the mechanisms underlying the observed disparities in unmet therapy needs, particularly those focused on how parental education influences both healthcare access and service utilization. Studies should also examine the role of health literacy, parental engagement, and social support in shaping these outcomes. Additionally, research should assess the effectiveness of interventions aimed at reducing these disparities. Longitudinal studies tracking children’s developmental trajectories in relation to therapy access and parental education could provide deeper insights into long-term effects and inform more targeted policy interventions. Finally, exploring the intersection of other factors, such as income, geographic location, and healthcare infrastructure, is important for better understanding and addressing the complex contributors to unmet therapy needs in children with developmental delays.

### Study Limitations

The current study highlights that a certain proportion of legal guardians do not provide information on the educational status of their households. The literature suggests that socially disadvantaged groups, in particular, are more prone to refuse to participate in surveys [42]. As a result, the identified inequalities in treatment status may be underestimated.

Other indicators of families’ socioeconomic status (SES), such as income or occupation, could not be considered because they were not collected as part of the SEE. Although education is a significant determinant of occupation and income, the literature indicates that education is independently related to child development and care utilization.

The data do not provide information on whether parents were first informed of their child’s developmental delay during the school entry examination, even though the timing of the diagnosis can greatly influence treatment decisions. As a result, the underlying reasons for the lack of treatment remain unexplored.

The data also do not provide information on the severity of developmental delay or the necessity of therapeutic intervention. The disparities found in unmet therapy needs for children with developmental delays may therefore be due not only to underprovision for children with low parental education but also to overprovision for children with high parental education.

### Conclusion

This investigation of German school entrants reveals high rates of unmet therapy needs related to developmental delays, with variation across different domains of child development. These unmet therapy needs affect children with lower parental education more frequently across all areas of developmental delay and are statistically significant for global developmental delays and delays in physical coordination, selective attention, visual‒motor skills, and language and speech. However, further studies are needed to explain the association between parental education and unmet therapy needs. The strong association between parental education and unmet therapy needs underscores the urgent need for substantial efforts to promote equal opportunities. Interventions must specifically address individual barriers to service utilization, structural barriers to service access, and factors related to service delivery to reduce inequalities in the unmet therapy needs of children with developmental delays.
